# Exosomal Transfer of miR-185 Is Controlled by hnRNPA2B1 and Impairs Re-endothelialization After Vascular Injury

**DOI:** 10.3389/fcell.2021.619444

**Published:** 2021-04-20

**Authors:** Yi Si, Fei Liu, Dongqing Wang, Chao Fang, Xiao Tang, Baolei Guo, Zhenyu Shi, Zhihui Dong, Daqiao Guo, Jianing Yue, Weiguo Fu

**Affiliations:** ^1^Department of Vascular Surgery, Zhongshan Hospital Fudan University, Shanghai, China; ^2^Department of Vascular and Endovascular Surgery, The First Affiliated Hospital of Zhengzhou University, Zhengzhou, China

**Keywords:** exosomes, miRNA transfer, hnRNPA2B1, re-endothelialization, vascular injury

## Abstract

Dysfunction of endothelial cells (ECs) contributes to restenosis after vascular reconstruction for patients with coronary artery disease (CAD). The intercellular communication between ECs and vascular smooth muscle cells (VSMCs) might be critical in the development of restenosis and can be mediated by exosomes carrying functional microRNAs. miR-185 is reported to be associated with atherosclerosis, whether it plays a similar role in restenosis is unknown. In this study, we observed an elevated level of extracellular miR-185 in platelet-derived growth factor (PDGF)-stimulated VSMCs. The medium from PDGF-stimulated VSMCs promoted miR-185 expression in rat aortic ECs and inhibited EC angiogenesis. PDGF-stimulated VSMCs transferred miR-185 into ECs via exosomes. Furthermore, we found that the CXCL12 gene, a target of miR-185, is essential for the angiogenic potential of ECs. Exosomes derived from miR-185 mimic transfected VSMCs attenuated re-endothelialization after vascular injury. Moreover, we show that exosome-mediated miR-185 transfer is modulated by hnRNPA2B1. We also observed that hnRNPA2B1 is up-regulated during neointima formation and hnRNPA2B1 inhibition accelerates re-endothelialization and attenuates neointima formation following carotid injury. Taken together, our results indicate that exosomal miR-185 transfer from VSMCs to ECs is controlled by hnRNPA2B1 and impairs re-endothelialization after vascular injury.

## Introduction

Vascular interventions, such as angioplasty and bypass, are often performed to correct coronary artery diseases such as atherosclerosis. However, the injury to the native vessel wall resulting from vascular intervention can cause an excessive wound healing response and vessel re-narrowing or restenosis (Suwanabol et al., 2011; Alraies et al., 2017). Restenosis after vascular reconstruction is attributed to several processes, including constrictive vessel remodeling, intimal hyperplasia, and retarded re-endothelialization (Guo et al., 2014). These processes contribute to excessive matrix protein production, an overgrowth of cells and matrix in the subintimal layer, and thrombosis (Inoue et al., 2011). The narrowing of the vessel lumen caused by intimal hyperplasia and constrictive remodeling can be ameliorated by rapid re-endothelialization. Vascular interventions used to correct atherosclerosis result in damage to the protective layer of endothelial cells (ECs) leaving smooth muscle cells (SMCs) exposed to the influence of cytokines and growth factors which then initiate intimal growth and the constructive remodeling of restenosis (Guo et al., 2014). Therefore, a method that can enable the rapid regrowth of the endothelial layer and selectively inhibit the proliferation of SMCs could prevent restenosis.

The cell-to-cell communication between ECs and SMCs is thought to be involved in the development of atherosclerosis and mediated by exosomes containing microRNAs (miRNAs) (Hergenreider et al., 2012; Zheng et al., 2017). Several miRNAs, such as miR-10a, miR-126, miR-145, miR-146a/b, miR-185, miR-210, and miR-326, are known to be expressed in plaques associated with atherosclerosis (Raitoharju et al., 2013). Some of these miRNAs are thought to modulate angiogenic and vessel stabilization through acting as communication molecules between SMCs and ECs (Zhou et al., 2013; Chen et al., 2015; Climent et al., 2015). It has been reported that this intercellular communication takes place by selectively packaging the regulators of SMCs, such as miR-143/145, into EC-derived exosomes, which are then delivered to SMCs in vessel walls (Hergenreider et al., 2012). Interestingly, it was recently shown that the miR-143/145 cluster, could be transferred from SMCs to ECs through the formation of tunneling nanotubes to modulate angiogenesis by reducing the proliferation of ECs and their capacity to form vessel-like structures *in vitro* through stimulation by transforming growth factor-β (TGF-β) (Climent et al., 2015).

Several studies have implicated miR-185 expression in the regulation of angiogenesis in ECs (Ho et al., 2012; Hou et al., 2016). Angiogenesis is inhibited in human microvascular ECs by miR-185, through the targeting and down-regulation of stromal interaction molecule 1 (STIM1) (Hou et al., 2016). The negative regulation of P2Y6 by miR-185, and the downstream ERK pathway, inhibits angiotensin II-induced human aortic vascular SMC (VSMC) proliferation (Wang et al., 2017). Furthermore, circulating miR-185 was proposed to be a novel biomarker for clinical outcome in patients with dilated cardiomyopathy as high miR-185 levels were associated with a more favorable prognosis as a consequence of repressing B cell function (Yu et al., 2016). These studies suggest that miR-185 has a potential modulatory role in cardiovascular diseases. Whether miR-185 plays a role in the progression of restenosis and SMC–EC communication in response to pathological conditions is still unknown.

Different algorithms, such as miRWalk, miRanda, and TargetScan 5.1, identify CXC motif ligand 12 (CXCL12), also known as stromal cell-derived factor 1 alpha (SDF-1α), as a target of miR-185. CXCL12 is known to enhance angiogenesis through CXCR7 activation in human umbilical vein ECs (Zhang M. et al., 2017). Additionally, the CXCL12/CXC motif receptor 4 (CXCR4) chemokine ligand/receptor axis can mediate the mobilization of SMC progenitors to promote injury-induced neointimal hyperplasia (Doring et al., 2014). CXCR4 is required for efficient re-endothelialization after vascular injury through endothelial wound healing and proliferation. Re-endothelialization is reduced and neointimal hyperplasia is enhanced after vascular injury when endothelial CXCR4 is deficient in atherosclerosis-prone mice (Noels et al., 2014).

To avoid degradation in an environment dominated by ribonucleases, miRNAs are packaged into exosomes. The exosomal miRNAs are released by ceramide-dependent secretion and transferred to recipient cells where they perform various functions including regulation, degradation, and cell-to-cell communication (Kosaka et al., 2010; Wojciechowska et al., 2017). Certain short sequence motifs are over-represented in the miRNA that are enriched in exosomes and are involved in the loading of these miRNAs into exosomes. Recently, the binding of sumoylated hnRNPA2B1 to miRNAs containing a GGAG motif was reported to be a mechanism for sorting miRNAs into T-cell-derived exosomes (Villarroya-Beltri et al., 2013). We observed that the EXO motif GGAG occurs in the miR-185 sequence listed in miRBase. Heterogeneous nuclear ribonucleoproteins (hnRNPs) are associated with the transcriptional and post-transcriptional modulation of gene expression. The modulation of SMC differentiation in stem cells and embryonic branchial arch artery development is thought to involve the direct binding of hnRNPA2B1 to the promoters of specific SMC genes such as *Sm*α*A* and *Sm22*α (Wang et al., 2012). More recently, hnRNPs have been shown to be major regulators of VSMC phenotype switching and arterial remodeling (Zhang L. et al., 2017).

In the present study, we assess whether miR-185 interacts with CXCL12 in EC angiogenesis and determine if exosome-mediated miR-185 transfer is modulated by hnRNPA2B1 in VSMCs stimulated with platelet-derived growth factor (PDGF). PDGF, a dimeric glycoprotein of subunits A and B, is known to stimulate cell growth and division, especially in the angiogenesis of blood vessel formation and in the proliferation of VSMCs (Patsch et al., 2015). In this study, we also observe if exosomes derived from miR-185 mimic transfected VSMCs could influence re-endothelialization and assess the expression of hnRNPA2B1 during neointima formation and re-endothelialization following carotid artery injury in a rat model using a balloon embolectomy catheter.

## Materials and Methods

### Cell Culture

Primary rat VSMCs were isolated from the aorta of an adult Sprague Dawley rat using a digestive method described previously (Ray et al., 2001). To obtain VSMCs, connective tissue, fat, and endothelium were first removed from the excised artery. The artery was then cut into 1-mm^2^ pieces and treated with 1 mL (1 mg/mL) of collagenase II (Sigma-Aldrich, St. Louis, MO, United States) and 0.125% trypsin for 1 h at 37°C. To harvest the cells, the digested tissue was centrifuged at 300 × *g* for 5 min. The cells were then cultured in Dulbecco’s Modified Eagle Medium (DMEM; Gibco, Grand Island, NY, United States) supplemented with 10% fetal bovine serum (FBS; Gibco), 100 IU/ml penicillin, and 100 μg/ml streptomycin. Passages 3–7 were used for experiments.

Primary rat aortic ECs were obtained by digesting aorta rings in serum-free DMEM containing 0.2% collagenase II as previously described (Kim et al., 2016). The tissue was shaken (100 rpm/min) for 30 min at 37°C. The supernatant was then collected, neutralized with 10% FBS, and centrifuged at 300 × *g* for 10 min. Harvested cells were cultured in Endothelial Cell Growth Medium-2 (Lonza, Basel, Switzerland) supplemented with 10% FBS. Passages 2–4 were used for experiments. HEK293 cells (Sigma-Aldrich) were cultured in DMEM containing 10% FBS at 37°C in 5% CO_2_.

### Cell Transfection

Endothelial cells that were grown to 50–60% confluence were transfected with miR-185 mimic/inhibitor or negative controls (100 nmol/L of each; GenePharma, Shanghai, China) using Lipofectamine 2000 (Invitrogen, Carlsbad, CA, United States) according to the manufacturer’s instructions. For miR-185 overexpression and knockdown in VSMCs, rat primary VSMCs were transfected with 80 nmol/L miR-185 mimic/inhibitor or negative controls using Lipofectamine 2000. The transfected cells were cultured for 48 h before use in subsequent experiments.

For hnRNPA2B1 knockdown, hnRNPA2B1-specific shRNA lentiviral particles (the shRNA target sequence is 5′–CAGGCGACAUAUUGGGAAATT–3′) or non-target shRNA control were purchased from GeneChem (Shanghai, China). VSMCs were transduced with hnRNPA2B1 shRNA carrying lentivirus for 6 h and then replaced with fresh medium for 48 h.

### Preparation of VSMC Conditioned Media (CM) and Cell Treatment

Vascular smooth muscle cells were grown in DMEM supplemented with 10% FBS until 70% confluent. VSMCs were serum-starved for 24 h and subsequently treated without (Basal) or with 20 ng/ml of PDGF-BB (PeProTech, NJ, United States) for 24 h. The media were collected and centrifuged at 3,000 × *g* for 15 min to remove whole cells and at 10,000 × *g* for 30 min to remove cell debris. The supernatant was designated as Basal-CM and PDGF-CM, respectively, and used to treat ECs. For RNase treatment, CM was pre-treated with or without 0.1% Triton X-100 for 5 min, then incubated with RNase A (10 μg/mL, Invitrogen) at 37°C for 15 min.

### Exosome Purification and Electron Microscopy

To isolate exosomes from the supernatant, an ExoQuick-TC Exosome Precipitation Solution (System Biosciences, Mountain View, CA, United States) was used according to the manufacturer’s instructions. Following purification, pelleted exosomes were fixed in 2% paraformaldehyde in phosphate-buffered saline (PBS), pH 7.4. The size and concentration of exosomes were analyzed by nanoparticle tracking analysis (NTA) with NanoSight NS300 (Malvern, United Kingdom). Transmission electron microscopy was used to confirm the presence of exosomes and observe their morphology as described previously (De Veirman et al., 2016). Western blot analysis was used to further characterize and verify the exosomes using the following exosome-specific biomarkers: CD63, CD81 (Abcam, Cambridge, United Kingdom), and HSP70 (Cell signaling technology). Ultracentrifugation (100,000 × *g* for 90 min) was performed to deplete VSMC-derived exosomes from the CM.

### Shuttling Assay for Cy3-Labeled miR-185

For transfection with Cy3-labeled miR-185 mimics, miR-185 mimics were first labeled using a Label IT siRNA Tracker Cy3 kit (Mirus, Madison, WI, United States) according to the manufacturer’s instructions. VSMCs were transfected with Cy3-labeled miR-185 mimics. The following day, cells were washed three times with PBS and cultured in fresh serum-free medium. After 24 h incubation, the culture medium was collected. Exosomes containing Cy3-miR-185 were purified from the medium and incubated with 2 μM PKH67 (Sigma-Aldrich) for 5 min at 25°C. They were washed four times using Amicon Ultra-0.5 (100 kDa, Millipore, Billerica, MA, United States), then stained with DAPI (Invitrogen) and visualized under an Eclipse TE2000 fluorescence microscope (Nikon, Tokyo, Japan).

### qRT-PCR

Total RNA was extracted from VSMCs, supernatant, exosomes, or ECs using an RNeasy mini kit (Qiagen, Hilden, Germany) according to the manufacturer’s instructions. HiScript Reverse Transcriptase (RNase H; Vazyme Biotech Co., Ltd., Nanjing, China) was used to synthesize cDNAs. Quantitative PCR was performed with SYBR Green Master Mix (Vazyme Biotech Co., Ltd) according to the manufacturer’s instructions using an ABI7900 Real-Time PCR System (Applied Biosystems, Foster City, CA, United States) and with the following primers: rno-miR-185, Forward 5′-TGCGCTCCCTGAGACCCTAACT-3′ and Reverse 5′-CCAGTGCAGGGTCCGAGGTATT-3′; U6, Forward 5′-CGCTTCGGCAGCACATATAC-3′ and Reverse 5′-AAATATGGAACGCTTCACGA-3′. Relative miRNA expression was normalized to U6 and quantified using the 2^–ΔΔ^ ^Ct^ method.

### Western Blotting

Exosome-derived proteins were extracted from exosomes using a Qproteome Mammalian Protein Prep Kit (Qiagen). Proteins from cell lysates were then analyzed using western blotting. Cells were first harvested and then suspended in RIPA lysis buffer (Beyotime, Haimen, China) containing 1 mM PMSF and a protease inhibitor cocktail. Protein content was quantified using a BCA Protein Assay Kit (Pierce, Rockford, IL, United States) and then separated with 10% SDS-PAGE. The proteins were transferred to polyvinylidene difluoride (PVDF) membranes (Millipore Corporation, Bedford, MA, United States), blocked with 5% BSA, and then incubated overnight at 4°C with the following primary antibodies: anti-hnRNPA2B1 (1:1,000; Abcam), anti-CXCL12 (1:1,000; Bioss, Beijing, China), anti-CXCR4 (1:100, Abcam) and anti-GAPDH (Santa Cruz Biotechnology, Santa Cruz, CA, United States). Membranes were then incubated with horseradish peroxidase (HRP)-conjugated secondary antibody (Abcam) for 1 h at room temperature. Enhanced chemiluminescent (ECL) reagent (Pierce, Rockford, IL, United States) was used to visualize the signals.

### Cell Proliferation

Endothelial cell proliferation was determined using a colorimetric BrdU kit (Roche Diagnostics, Mannheim, Germany). Briefly, cells (5 × 10^3^/well) were seeded into 96-well plates in triplicate. After growing to 70–80% confluence, the cells were serum-starved for 24 h and subsequently treated with CM (CM-fresh medium, 1:1) or exosomes (100 μg/mL) for 24 h. BrdU was added for the last 2 h of treatment. The absorbance at 450 nm was measured using a Microplate reader (Thermo).

### Scratch Wound Healing Assay

Cell migration was assessed by using a scratch wound healing assay. ECs were grown to 80–90% confluence in six-well plates. Cells were treated with 2 mM thymidine (an inhibitor of DNA synthesis, Sigma-Aldrich) and a wound was scraped using a sterilized 200-μL pipette tip. Cells were washed with PBS and then incubated with CM (CM-fresh medium, 1:1) or exosomes (100 μg/mL) for 24 h. Images of cell migration were captured by a phase-contrast microscope and used to calculate the healing rates.

### Matrigel Angiogenesis Assay *in vitro*

To measure tube formation, cells (1.5 × 10^4^) were first seeded on top of Matrigel (BD Biosciences, San Jose, CA, United States) in a 24-well plate. The cells were then treated with CM (CM-fresh medium, 1:1) or exosomes (100 μg/mL) for 24 h. The cumulative tube length of the network structure was estimated from images by randomly selecting five microscopic fields (4 × magnification) taken on a phase-contrast microscope (Olympus) and quantified using ImageJ software.

### Dual-Luciferase Reporter Assay

A fragment of CXCL12 3′ UTR containing either wildtype (wt) or mutant (mut) miR-185 binding sites was synthesized. The fragment was inserted into a pmirGLO vector (Promega, Madison, WI, United States) and 200 ng of the wt or mut construct was co-transfected in HEK293 cells with either the vehicle control, the miR-185 mimic/inhibitor, or negative control (50 nM) using Lipofectamine 2000 reagent (Invitrogen) according to manufacturer’s instructions. After 48 h, the resulting firefly and Renilla luciferase activities were measured using the Dual-Glo^TM^ Luciferase Assay system (Promega). Relative luciferase activity was normalized to Renilla luciferase activity.

### Rat Carotid Injury Model

The balloon-induced vascular injury model was performed as described in detail by Tulis (2007). In brief, male Sprague Dawley rats (250–300 g) obtained from the SLAC Laboratory Animal Co., Ltd. (Shanghai, China), were first administered 75 mg/kg pentobarbital and 100 U/kg heparin sodium. Once anesthetized and heparinized, a two French Fogarty arterial embolectomy balloon catheter (Edwards Lifesciences, Irvine, CA, United States) was introduced through the left external carotid artery. To induce injury, the balloon catheter was distended and deflated with saline three times. The same procedure was replicated in sham-operated rats without catheterization.

For the systematical delivery of exosomes, rats were injected with exosomes (20 μg) intravenously twice over 1 week. For the delivery of lentiviral hnRNPA2B1 shRNA, 50 μL of solution containing either lentiviral control or hnRNPA2B1-specific shRNA were infused into the rats at the injured site immediately post-injury. To examine whether miRNA-185 is involved in the effects of hnRNPA2B1 on re-endothelialization, 50 μL of solution containing lentiviral hnRNPA2B1-specific shRNA and lenti-miR-185 (GenePharma) were infused into the rats at the injured site. After 30 min incubation, the solution was withdrawn and the left external common carotid artery was ligated. Blood flow was then restored and the rats were left undisturbed for 7 or 14 days.

### Tissue Preparation and Morphometric Analysis

Animals were euthanized by intoxication with 100% carbon dioxide. Carotid arteries were then harvested and fixed overnight in 4% paraformaldehyde dissolved in PBS. The fixed tissues were then embedded in paraffin. After deparaffinization and rehydration, 4 μm thick sections were stained with hematoxylin and eosin. The mean level of neointima formation was determined from intima to media ratios (I/M) of five independent artery sections using ImageJ software.

### Immunofluorescence Staining

Antigens were first retrieved from arterial sections using a 5-min high-pressure process (100 × sodium citrate buffer, pH 6.0). The arteries were then blocked in PBS with 10% goat serum for 1 h and incubated at 4°C overnight with the following primary antibodies: hnRNPA2B1 (1:200; Abcam), a-SMA (1:100; Abcam), and CD31 (1:50; Abcam). The PBS-washed sections were then incubated with the fluorescein-conjugated secondary antibody for 1 h at 37°C. DAPI was used to stain nuclei before images were obtained using a fluorescence microscope (Olympus DX51, Japan).

### Re-endothelialization Assay

Re-endothelialization of the carotid artery was determined by a procedure using Evan’s blue dye (Sigma-Aldrich). Staining of the arteries occurred 7 days after the balloon injury in rats. Anesthetized animals were injected with 50 μL of 5% Evan’s blue dye into the tail vein, followed by fixation with perfusion of 10% formalin. Rats were euthanized and the relevant segments of carotid arteries were harvested and observed under a light microscope. The ratio between the area stained blue and the total carotid artery area was used to determine the damage to the endothelial lining.

### Statistical Analysis

At least three independent experiments were used to obtain data from *in vitro* experiments and six samples or more were used from each group for *in vivo* studies. Results are presented as the means ± SD unless otherwise stated. Statistical analyses were performed using a two-tailed Student *t*-test or one-way ANOVA and calculated using SPSS 17.0 software. *P* < 0.05 was considered statistically significant.

## Results

### Conditional Medium From PDGF-Stimulated VSMCs Induces the Expression of miR-185 in ECs and Attenuates Angiogenesis

**FIGURE 1:**
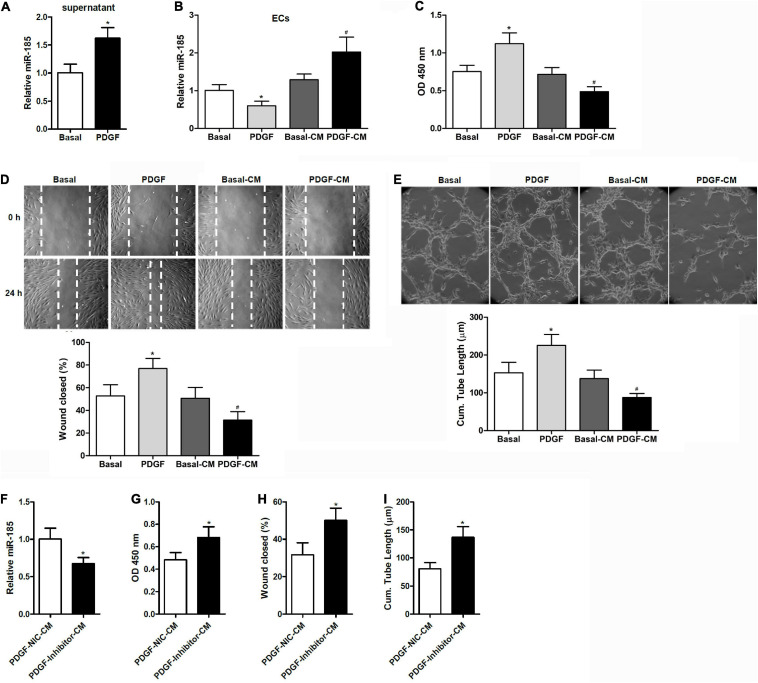
Conditional medium derived from VSMCs stimulated with PDGF induces miR-185 expression in ECs and attenuates angiogenesis. **(A)** Levels of miR-185 in the supernatant of VSMCs under basal conditions (Basal) or exposed to PDGF-BB (20 ng/ml) (PDGF) for 24 h. **(B)** Levels of miR-185 in ECs exposed to different media for 24 h were determined by qRT-PCR assays. Basal, basal medium; PDGF, PDGF treatment (20 ng/ml); Basal-CM, medium from VSMCs in basal conditions; PDGF-CM, medium from VSMCs after PDGF stimulation. **(C)** Proliferation of ECs exposed to different media for 24 h as assessed by a BrdU assay. **(D)** Migration of ECs exposed to different media for 24 h assessed by a scratch wound assay. Migration is expressed as the relative recovery rate of the scratch wound. **(E)** Angiogenesis of ECs exposed to different media for 24 h was assessed by a tube formation assay. The quantitative analysis of the tube lengths is shown. *P < 0.05 vs. Basal, ^#^*P* < 0.05 vs. Basal-CM. **(F–I)** ECs were exposed to media from SMCVs stimulated with PDGF transfected with miR-185 inhibitor or its negative control for 24 h. Levels of miR-185 in ECs **(F)**, cell proliferation **(G)**, cell migration **(H)**, and tube formation **(I)** of ECs. *P < 0.05.

To assess the influence of PDGF stimulation on miR-185 expression, VSMCs were grown in the presence of PDGF-BB for 24 h. Levels of miR-185 assessed by qRT-PCR were elevated significantly in the supernatant of PDGF-stimulated VSMCs compared to the basal-level expression found in unstimulated cells (Figure 1A). Results were also obtained for other miRNAs involved in re-endothelialization including pro-angiogenic miRNAs miR-126, miR-17, miR-130a, miR-210, and anti-angiogenic miRNAs miR-221, miR-222, miR-92a, and miR-214 (Qin and Zhang, 2011; Supplementary Figure 1). Similar results were found with pro-angiogenic miR-210 although the opposite occurs with miR126a-3p and miR126a-5p.

When ECs were exposed to media from VSMCs stimulated with PDGF-BB (PDGF-CM), the level of miR-185 expression increased significantly and cell proliferation was reduced (Figures 1B,C). This was not observed with media from cells grown without PDGF (Basal-CM), which indicates that medium from PDGF-stimulated VSMCs could significantly influence the proliferation of ECs. The ability of ECs to migrate was assessed by a wound assay after exposure to different media for 24 h. Wound healing ability was significantly reduced in the ECs grown in PDGF-stimulated VSMC media compared with those grown in basal VSMC media or the control (Figure 1D). The angiogenesis of ECs was assessed by a tube formation assay. Similar to the results of the wound closure assay, tube length was found to be reduced in ECs grown in the media of PDGF-stimulated VSMCs (Figure 1E). ECs were then exposed to media from PDGF-stimulated VSMCs transfected with a miR-185 inhibitor or a negative control. Although Levels of miR-185 expression were reduced in ECs grown in media from VSMCs transfected with a miR-185 inhibitor, levels of proliferation, migration, and tube formation were increased (Figures 1F–I). Overall, these results reveal that CM derived from PDGF-stimulated VSMCs increases the expression of miR-185 in ECs and attenuates angiogenesis whereas the same media from PDGF-stimulated VSMCs with miR-185 inhibited does not attenuate angiogenesis.

### Transfer of miR-185 From PDGF-Stimulated VSMCs Into ECs Is via Exosomes

**FIGURE 2:**
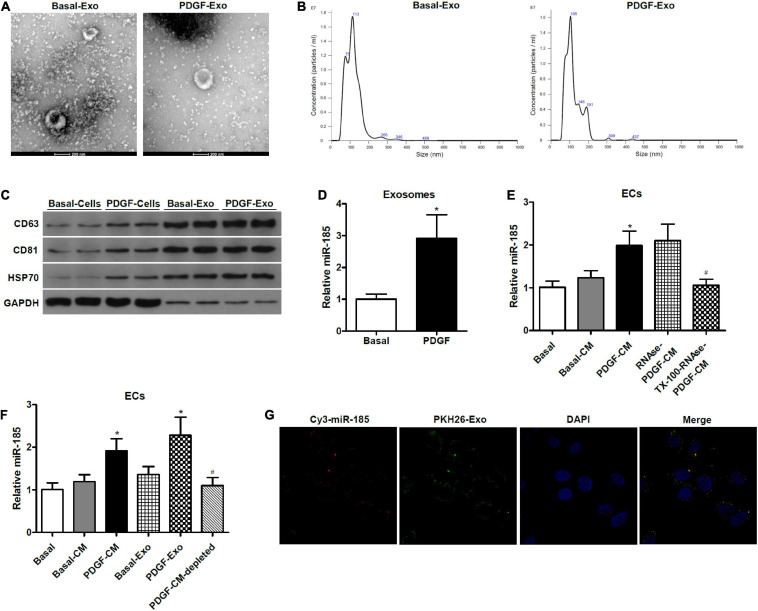
miR-185 was transferred from PDGF-stimulated VSMCs to ECs via exosomes. **(A)** Representative electron microscopy image of isolated exosomes from the supernatant of VSMCs under both basal (Basal-Exo) and PDGF conditions (PDGF-Exo). **(B)** Representative NTA histogram of isolated exosomes from the supernatant of VSMCs under both basal (Basal-Exo) and PDGF conditions (PDGF-Exo). **(C)** Western blot analysis of exosome proteins. **(D)** qRT-PCR detection of miR-185 expression in exosomes derived from VSMCs in basal condition and from VSMCs exposed to 20 ng/mL PDGF for 24 h. *P < 0.05. **(E)** Levels of miR-185 in ECs incubated with CM from VSMCs in basal conditions and from VSMCs exposed to 20 ng/mL PDGF were treated with or without RNase I and/or Triton X-100. *P < 0.05 vs. Basal-CM, ^#^*P* < 0.05 vs. RNAse-PDGF-CM. **(F)** Levels of miR-185 in ECs exposed to different media or exosomes derived from VSMCs following different treatment. *P < 0.05 vs. Basal-CM or Basal-Exo, ^#^*P* < 0.05 vs. PDGF-Exo. **(G)** Internalization of PKH67-labeled exosomes derived from VSMCs transfected with Cy3-miR-185.

Electron microscopy was used to obtain images of exosomes that were purified from supernatant collected from VSMCs (Figure 2A). The size and concentration of isolated exosomes were measured by nanoparticle tracking analysis (NTA), which showed that exosomes from VSMCs both in basal conditions or after treatment with PDGF have a similar in number and size-distribution within the typical range for exosomes (Figure 2B). The exosome-related protein markers CD63, CD81, and HSP70 were analyzed in VSMCs and exosomes by western blotting (Figure 2C). Levels of CD63, CD81, and HSP70 were increased in the exosomes purified from VSMC supernatant. In addition, qRT-PCR revealed a higher expression of miR-185 in exosomes derived from VSMCs exposed to PDGF than from VSMCs in basal conditions (Figure 2D).

To determine whether constituents secreted by the VSMCs were responsible for the increase of miR-185 in the ECs and to characterize the form of miR-185 transmission, we exposed the VSMC-CM to ribonuclease (RNase), with or without pretreatment of the phospholipid membrane disruptor Triton X-100. The ability of the VSMC-CM to elevate levels of miR-185 in ECs was unaffected by RNase, but treatment with Triton X-100 before RNase prevented this increase (Figure 2E), suggesting that exosome-protected miR-185 are transmitted to ECs. To further establish if miR-185 from VSMCs is transferred into ECs via exosomes, ultracentrifugation was performed to deplete exosomes in the supernatant from PDGF-stimulated VSMCs. The levels of miR-185 increased significantly in ECs exposed to PDGF-CM or exosomes derived from PDGF-stimulated VSMCs (PDGF-Exo). In exosome-depleted supernatants, miR-185 levels were decreased compared with PDGF-CM treated ECs, and no difference was observed in ECs grown in basal conditions (Figure 2F). Confocal microscopy was used to analyze the internalization of PKH67-labeled exosomes derived from VSMCs transfected with Cy3-miR-185. The results showed that exosomes were taken up by the ECs and the Cy3-miR-185 signals and the PKH67 signals were co-localized in the cytoplasm of ECs (Figure 2G). This indicates that the process by which miR-185 is transferred from PDGF-stimulated VSMCs to ECs is via exosomes.

### miR-185 Inhibits EC Angiogenesis by Targeting CXCL12

**FIGURE 3:**
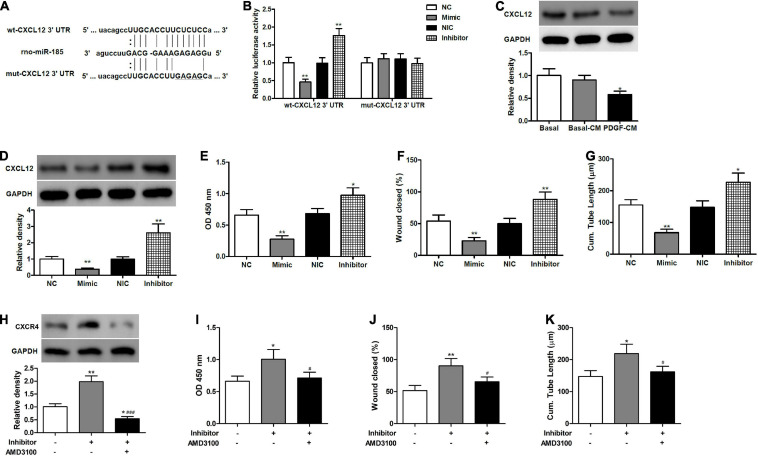
miR-185 directly targets CXCL12 in ECs. **(A)** The predicted miR-185 target site in the 3′ UTR of CXCL12. **(B)** HEK293 cells were co-transfected with miR-185 mimic or inhibitor together wildtype (wt)- or mutant (mut)-CXCL12 3′ UTR construct. Twenty-four hours later, cells were harvested for the determination of luciferase activities. **P < 0.01 vs. NC or NIC group. **(C)** Western blot analysis of CXCL12 in ECs treated with different media. *P < 0.05 vs. Basal-CM. **(D)** Western blot analysis of CXCL12 in ECs transfected with miR-185 mimics and inhibitor for 48 h. **(E)** Proliferation of ECs transfected with miR-185 mimics and inhibitor was assessed by a BrdU assay. **(F)** Migration of ECs transfected with miR-185 mimics and inhibitor was assessed by a scratch wound assay. Quantification of migration is expressed as a relative rate of scratch wound healing. **(G)** Angiogenesis of ECs transfected with miR-185 mimics and inhibitor was assessed by a tube formation assay. Quantitative analysis of the tube lengths is shown. *P < 0.05, **P < 0.01 vs. NC or NIC group. **(H–K)**. ECs transfected with miR-185 inhibitor were treated with AMD3100 (5 μM) for 24 h. Western blot analysis of CXCR4 **(H)**, proliferation **(I)**, migration **(J)**, and angiogenesis **(K)** of ECs transfected with miR-185 inhibitor and treated with AMD3100 were assessed. *P < 0.05, **P < 0.01 vs. Blank, ^#^*P* < 0.05, ^###^*P* < 0.001 vs. Inhibitor.

CXCL12 serves as a key regulator of angiogenesis and plays an important role in re-endothelialization and neointimal hyperplasia after vascular injury (Subramanian et al., 2010; Yin et al., 2010). In the present work, different algorithms, such as miRWalk, miRanda, and TargetScan 5.1, predicted CXCL12 to be a target of miR-185. Therefore, we aimed to examine whether miR-185 functions through CXCL12 to inhibit EC angiogenesis. We mutated the miR-185 target site in the 3′ UTR of CXCL12 (Figure 3A) and then compared the luciferase activity in HEK293 cells co-transfected for 24 h with miR-185 mimic or inhibitor together with wt- or mut-CXCL12 3′ UTR construct (Figure 3B). Luciferase activity was reduced in cells transfected with wt-CXCL12 3′ UTR and miR-185 mimic. In contrast, luciferase activity increased in cells transfected with wt-CXCL12 3′ UTR in the presence of an inhibitor. However, no changes in luciferase activity were observed with mut-CXCL12 3′ UTR in the presence of the mimic miR-185 or the inhibitor compared with the control. The protein levels of CXCL12 in the ECs treated with different media or transfected with miR-185 mimics or inhibitor were assessed by western blotting (Figures 3C,D). CXCL12 protein levels were significantly lower in cells treated with PDGF-stimulated VSMC media and in cells transfected with a miR-185 mimic but were significantly higher when transfected with a miR185 inhibitor. Proliferation, migration, and angiogenesis were assessed in ECs transfected with a miR-185 mimic or inhibitor (Figures 3E–G). The miR-185 mimic inhibited all three processes whereas the miR-185 inhibitor significantly increased proliferation, migration, and angiogenesis. These results confirm that miR-185 directly targets CXCL12 in ECs and both influence proliferation, migration, and angiogenesis. To confirm these results we performed a rescue experiment by blocking CXCR4 function with inhibitors (AMD3100) in miR-185 inhibitor transfected cells (Figures 3H–K). Levels of CXCR4 were increased with a miR-185 inhibitor but reduced in the presence of AMD3100. The proliferation, migration, and angiogenesis of ECs transfected with miR-185 inhibitor were all increased but reduced to basal levels in cells treated with AMD3100. These results confirm that miR-185 inhibits EC angiogenesis by mediating the CXCL12/CXCR4 axis.

### Exosomal miR-185 Derived From VSMCs Impairs Re-endothelialization in Balloon-Injured Carotid Artery

**FIGURE 4:**
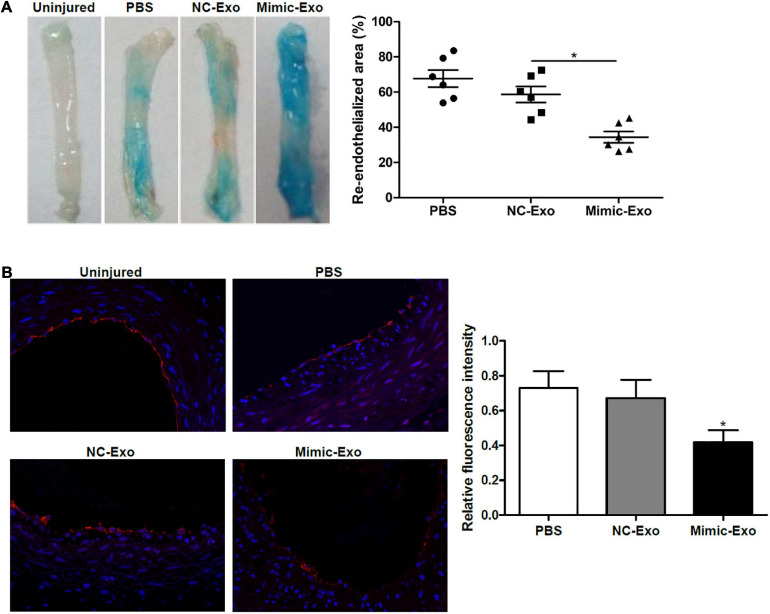
Exosomal miR-185 derived from VSMCs attenuates re-endothelialization in balloon-injured rat carotid arteries. **(A)** Representative images of Evan’s blue staining of carotid arteries from rats treated with exosomes derived from miR-185 mimic transfected VSMCs at day 7 after balloon injury. The percent area of re-endothelialization was quantified as white-area/total-area ratio. **(B)** Immunofluorescence staining for CD31 in the sections at 1 week after injury. The relative fluorescence intensity is shown. *P < 0.05 vs. NC-Exo.

After determining that miR-185 could possibly attenuate angiogenesis *in vitro* we next assessed the exosomal delivery of exosomal miR-185 derived from VSMCs *in vivo* using a rat model of carotid artery injury. Seven days after balloon injury, the rats were treated with exosomes derived from miR-185 mimic transfected VSMCs. Representative images of Evan’s blue staining of carotid arteries can be seen in Figure 4A. The absence of Evan’s blue staining indicated that the endothelial layer is intact whereas vessels stained entirely blue indicated the complete loss of the endothelial lining. The percent area of re-endothelialization was quantified as the white-area/total-area ratio. Rats treated with exosomes derived from miR-185 mimic transfected VSMCs had significantly decreased re-endothelialization following the injury (Figure 4A). Immunofluorescence staining for CD31, an endothelial cell marker used to quantify angiogenesis, was significantly less intense in the sections of artery from rats that received exosomes derived from miR-185 mimic transfected VSMCs (Figure 4B). These results indicate that exosomal miR-185 derived from VSMCs attenuates re-endothelialization in balloon-injured rat carotid arteries.

### Exosomal miR-185 Transfer From VSMCs to ECs Is Controlled by hnRNPA2B1

**FIGURE 5:**
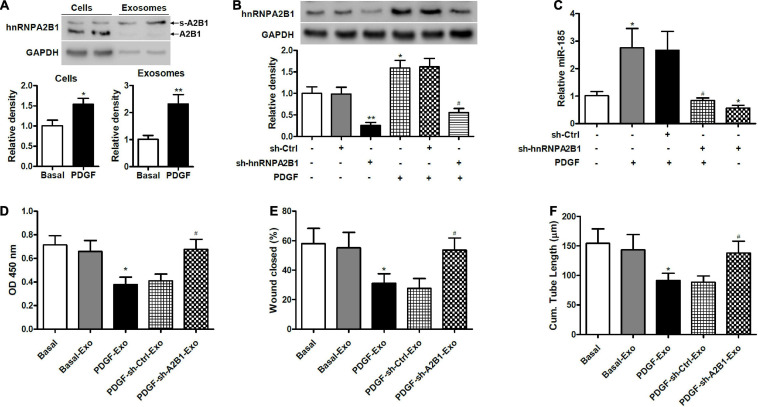
Exosomal miR-185 transfer from VSMCs to ECs is modulated by hnRNPA2B1. **(A)** Western blot analysis of hnRNPA2B1 in VSMCs in basal condition and from VSMCs exposed to 20 ng/mL PDGF for 24 h and their exosomes. A2B1: hnRNPA2B1; s-A2B1: sumoylated hnRNPA2B1. *P < 0.05, **P < 0.01. **(B)** Expression of hnRNPA2B1 in VSMCs transfected with control shRNA (sh-Ctrl) or hnRNPA2B1 shRNA (sh-hnRNPA2B1) carrying lentivirus for 24 h and then stimulated with PDGF for 24 h. *P < 0.05, **P < 0.01 vs. control group, ^#^*P* < 0.05 vs. PDGF group. **(C)** Expression of miR-185 in exosomes derived from VSMCs transduced with control shRNA (sh-Ctrl) or hnRNPA2B1 shRNA (sh-hnRNPA2B1) carrying lentivirus for 24 h and then stimulated with or without PDGF for 24 h. *P < 0.05 vs. control group, ^#^*P* < 0.05 vs. PDGF group. **(D)** Proliferation of ECs treated with exosomes derived from VSMCs transduced with control shRNA (sh-Ctrl) or hnRNPA2B1 shRNA (sh-hnRNPA2B1) carrying lentivirus for 24 h and then stimulated with or without PDGF was assessed by a BrdU assay. **(E)** Migration of ECs treated with exosomes derived from VSMCs transfected with control shRNA (sh-Ctrl) or hnRNPA2B1 shRNA (sh-hnRNPA2B1) carrying lentivirus for 24 h and then stimulated with or without PDGF was assessed by a scratch wound assay. Quantification of migration is expressed as the relative rate of scratch wound healing. **(F)** Angiogenesis of ECs treated with exosomes derived from VSMCs transfected with control shRNA (sh-Ctrl) or hnRNPA2B1 shRNA (sh-hnRNPA2B1) carrying lentivirus for 24 h and then stimulated with or without PDGF was assessed by a tube formation assay. Quantitative analysis of the tube lengths is shown. *P < 0.05 vs. Basal group, ^#^*P* < 0.05 vs. PDGF-Exo group.

To determine if hnRNAPA2B1 was involved in the selective transfer of miR-185, we used western blot analysis to assess the levels of hnRNPA2B1 in VSMCs and exosomes under basal conditions or exposed to PDGF for 24 h (Figure 5A). Levels of hnRNPA2B1 were elevated in cells and exosomes treated with PDGF compared with those grown without PDGF. In addition, the molecular weight of hnRNPA2B1 from exosomes was higher, which is consistent with a report by Villarroya-Beltri et al. (2013) and suggests that hnRNPA2B1 might be sumoylated in exosomes (Villarroya-Beltri et al., 2013). Levels of hnRNPA2B1 were then assessed in VSMCs transduced with control shRNA or hnRNPA2B1 shRNA carrying lentivirus and stimulated with or without PDGF (Figure 5B). The inhibition of hnRNPA2B1 was confirmed by a significant reduction in hnRNPA2B1 and treatment with PDGF significantly increased levels of hnRNPA2B1 except when hnRNPA2B1 is silenced. We then assessed the expression of miR-185 in exosomes derived from VSMCs transduced with control shRNA or hnRNPA2B1 shRNA carrying lentivirus and then stimulated with or without PDGF (Figure 5C). The expression of miR-185 was significantly increased in cells treated with PDGF, silencing of hnRNPA2B1 blocked the increases of miR-185 levels induced by PDGF and reduced miR-185 levels in the absence of PDGF. The proliferation, migration, and angiogenesis of ECs treated with exosomes derived from VSMCs transduced with control shRNA or hnRNPA2B1 shRNA carrying lentivirus and stimulated with or without PDGF was assessed (Figures 5D–F). Levels of proliferation, migration, and angiogenesis in ECs treated with exosomes derived from PDGF-treated VSMCs were significantly lower than in ECs grown under basal conditions. However, when hnRNPA2B1 is silenced the levels of proliferation, migration, and angiogenesis in ECs treated with exosomes derived from PDGF-treated VSMCs are the same as the basal levels. These results provide evidence that exosomal miR-185 transfer from VSMCs to ECs is modulated by hnRNPA2B1.

### hnRNPA2B1 Inhibition Promotes Re-endothelialization and Reduces Neointima Formation Induced by Balloon Injury

**FIGURE 6:**
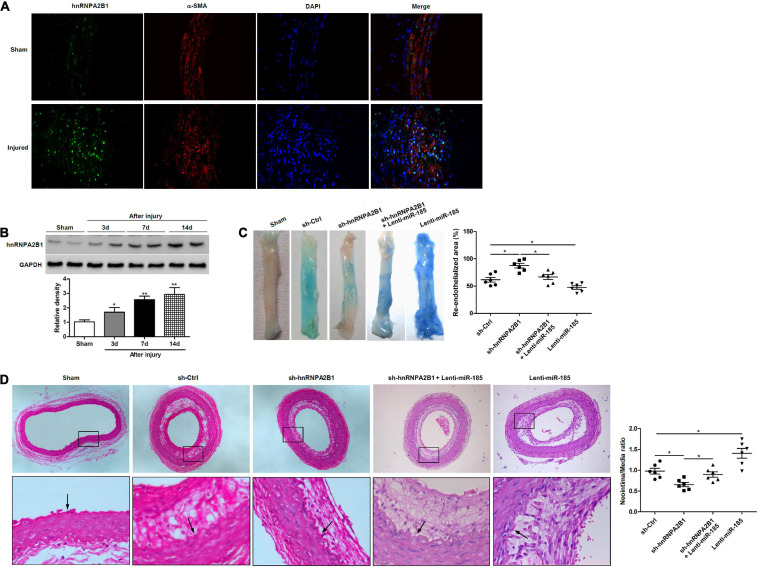
hnRNPA2B1 inhibition promotes re-endothelialization and reduces neointima formation following carotid injury. **(A)** Immunofluorescence staining shows hnRNPA2B1 (green) expression and localization in the arteries post-injury. **(B)** Levels of hnRNPA2B1 expression were determined in uninjured rat carotid arteries and at different times after left carotid arterial balloon injury by western blotting. *P < 0.05, **P < 0.01 vs. Sham group. **(C)** Representative images of Evan’s blue staining of carotid arteries from rats treated with control shRNA (sh-Ctrl), hnRNPA2B1 shRNA (sh-hnRNPA2B1) carrying lentivirus, sh-hnRNPA2B1 and Lenti-miR-185, or Lenti-miR-185 at day 7 after balloon injury. **(D)** Representative images of hematoxylin and eosin-stained paraffin sections of carotid arteries from rats treated with control shRNA (sh-Ctrl), hnRNPA2B1 shRNA (sh-hnRNPA2B1) carrying lentivirus, sh-hnRNPA2B1 and Lenti-miR-185, or Lenti-miR-185 at day 14 after balloon injury. Arrows indicate the internal elastic lamina. Quantifications of neointimal formation as intima/media area ratio are shown. *P < 0.05.

To determine how hnRNPA2B1 influenced re-endothelialization and neointima formation *in vivo*, we assessed levels of hnRNPA2B1 expression in injured rat carotid arteries. Immunofluorescence staining revealed higher levels of immunofluorescence staining hnRNPA2B1 are detectable in arteries post-balloon injury (Figure 6A). The expression of hnRNPA2B1 in injured rat carotid arteries appears to increase from day 3 and then remain at elevated levels (Figure 6B). Representative images of Evan’s blue staining of carotid arteries from rats treated with control shRNA (sh-Ctrl), hnRNPA2B1 shRNA (sh-hnRNPA2B1) carrying lentivirus, sh-hnRNPA2B1 and Lenti-miR-185, or Lenti-miR-185 at day 7 after balloon injury revealed that greater re-endothelialization occurs in the arteries with hnRNPA2B1 silenced whereas the least occurs when miR-185 is overexpressed (Figure 6C). The quantification of neointimal formation determined from hematoxylin and eosin-stained paraffin sections of carotid arteries from rats treated with sh-Ctrl, sh-hnRNPA2B1 carrying lentivirus, sh-hnRNPA2B1 and Lenti-miR-185, or Lenti-miR-185 14 days after balloon injury show that the neointima/media ratio is lower in rats when hnRNPA2B1 is silenced but is higher when miR-185 is overexpressed (Figure 6D). To conclude, hnRNPA2B1 inhibition promotes re-endothelialization and reduces neointima formation following carotid injury.

## Discussion

Restenosis after vascular reconstruction can occur as the consequence of two major processes intimal hyperplasia and retarded re-endothelialization. Intimal hyperplasia is the thickening of the tunica intima of a blood vessel, which involves the recruitment and proliferation of VSMCs. Whereas, retarded re-endothelialization is associated with the dysfunction of ECs. Exosomes containing miRNA have recently emerged as important players in the intercellular communication between ECs and VSMCs (Hergenreider et al., 2012; Zheng et al., 2017). The ability to control restenosis with miRNAs through the use of exosomes is gaining interest (Pashova et al., 2020), but the exact mechanisms, such as the involvement of hnRNPs, are not clearly understood (Huber and Holvoet, 2015; Kapustin and Shanahan, 2016; Mukherjee et al., 2016). In this study, we investigated the ability of exosomes to mediate the delivery of miR-185 from VSMCs to ECs and assessed their effects on endothelialization after vascular injury. We selected miR-185 because it is reported to be associated with atherosclerosis, involved in the regulation of angiogenesis in ECs, and suppresses proliferation in dysfunctional human aortic VSMCs. Moreover, the EXO motif GGAG, which binds to sumoylated hRNPA2B1, occurs in the miR-185 sequence. Consequently, we also determined the possibility that hnRNPA2B1 modulated exosome-mediated miR-185 transfer. Furthermore, we discovered that CXCL12, which encodes a chemokine protein involved in angiogenesis, was a potential target of miR-185 and included the impact of this gene on re-endothelialization after endothelial damage.

We found that conditional media derived from PDGF-stimulated VSMCs induces miR-185 in ECs and attenuates angiogenesis whereas media from PDGF-stimulated VSMCs with miR-185 silenced reduces the inhibitory effect on wound healing, tube formation, and proliferation in ECs. Previous studies have reported that miR-185-5p was a key regulator of angiogenesis through targeting cathepsin K (CatK), which plays an important role in regulating vascular repair (Li et al., 2019). Wei and Zhao (2020) reported that miR-185 inhibits angiogenesis in human ovarian microvascular endothelial cells by downregulating the level of VEGFA (Wei and Zhao, 2020). In this study, the supernatant from VSMCs was confirmed to contain exosomes that expressed miR-185. These results are supported by a recent study, which reports that extracellular vesicles secreted by atherogenic macrophages transfer miRNA that have the capability of inhibiting cell migration (Nguyen et al., 2018). The authors of the study claim that miR-185 was one of several miRNAs isolated from atherogenic exosomes and that these extracellular vesicles are taken up by recipient macrophages. Furthermore, silencing the most abundant of these miRNAs, miR-146a, prevented exosomes from being transferred to macrophages and reduced the inhibitory effect on macrophage migratory capacity. There is substantial evidence supporting the role of extracellular vesicle (EV)- delivered microRNAs in communication between ECs and SMCs (Zhou et al., 2013; Gu et al., 2017; Jansen et al., 2017). Gu et al. (2017) reported that EVs could transfer miR-195 from ECs to SMCs to inhibit the expression of the serotonin transporter (5-HTT) and the proliferation of SMCs (Gu et al., 2017). Jansen et al. (2017) revealed that EV- delivered miR-126 transfer from ECs to SMCs reduced VSMC proliferation and subsequent neointima formation after vascular injury (Jansen et al., 2017). Our *in vitro* observations of miR-185 were confirmed *in vivo*. Exosomes derived from miR-185 mimic transfected VSMCs attenuated re-endothelialization after endothelial damage in a rat model of carotid artery injury created by balloon embolectomy catheter.

To learn more about the pathways or genes influenced by miR-185, we searched miRWalk, miRanda, and TargetScan 5.1 and identified CXCL12 as a potential target gene. CXCL12 was found to be essential for the angiogenic potential of ECs. CXCL12 protein levels were lower in cells treated with PDGF-stimulated VSMC media and on transfection with a miR-185 mimic but were significantly higher when transfected with a miR-185 inhibitor. CXCL12 and its receptor CXCR4 direct the recruitment of smooth muscle progenitor cells in neointima formation after vascular injury (Subramanian et al., 2010), and play a similar role in the mobilization and recruitment of EPCs after vascular injury (Yin et al., 2010). CXCL12 and its receptor CXCR4 stimulate several intracellular signaling cascades, in particular, the activation of Ras on the plasma membrane which controls cell proliferation and differentiation (Zhao et al., 2006). The activation of c-Raf, mitogen-activated protein kinase kinase (MAPKK or MEK1/2) by the CXCL12/CXCR4 axis, in turn, activates extracellular signal-regulated kinase (ERK1/2), allowing ERK1/2 to enter the nucleus, where it instigates several transcription factors. Moreover, CXCL12/CXCR4-induces CyPA phosphorylation and translocation to the nucleus. Interestingly, CyPA forms a complex with hnRNPA2 in the nucleus. CXCL12/CXCR4/CyPA mediates the nuclear export of hnRNPA2, activation and nuclear translocation of ERK1/2, and chemotactic cell migration and proliferation (Pan et al., 2008).

Although exosomes are known to contain miRNAs, the processes by which miRNAs are sorted into exosomes are largely unknown. In this study, in addition to verifying the possible interactions of miR-185 on CXCL12 through mutating the putative 3′ UTR binding site, we also provide evidence that exosome-mediated miR-185 transfer is modulated by hnRNPA2B1 because an shRNA against hnRNPA2B1 suppressed the transfer of miR-185 from VSMCs to ECs. Furthermore, hnRNPA2B1 is up-regulated during neointima formation and hnRNPA2B1 inhibition accelerates re-endothelialization and attenuates neointima formation following carotid injury.

The abnormal expression of miRNAs is thought to be associated with several diseases in humans (Mendell and Olson, 2012). The importance of their regulation by hnRNPs, which involves both positive and negative feedback mechanisms, is becoming realized (Richard et al., 2017; Sun et al., 2017). For instance, HuR, a negative regulator of miRNA function, is thought to accelerate the exportation of exosome-mediated miRNAs from human cells by reversibly binding miRNAs to replace them in complexes with Argonaute proteins (Mukherjee et al., 2016). From the thousands of miRNAs that have been identified only a few hundred are thought to be packaged in exosomes. Discovering the factors that are involved in the selection and exportation of these specific miRNAs would improve the ability to exploit exosomes in the treatment of diseases. One study used a cell-free system and a miRNA that was abundant in exosomes, miR-233, to discover that a protein called Y-box Protein 1 (YBX1) was required to selectively package miR-233 into exosomes (Shurtleff et al., 2016). In our study, miR-185 seems to be selected by the sorting motif GGAG, which is recognized by hnRNPA2B1, however, no such motif exists in miR-233. Therefore, either other factors may be involved in the selection of exosomal miRNA or different mechanisms are associated with specific miRNAs. This area is worthy of further investigation. For instance, is there a possibility that HuR, CyPA, or YBX1 are involved in the exosomal regulation or transfer of miR-185. Moreover, would mutating the GGAG motif influence the interaction between miR-185 and hnRNPA2B1.

## Conclusion

In this study, we determined that miR-185 was transferred into ECs by exosomes through PDGF-stimulated VSMCs. CXCL12, a target of miR-185, is essential for the angiogenic potential of ECs. Furthermore, the exosome-mediated transfer of miR-185 was found to be modulated by hnRNPA2B1 because the transfer of miR-185 from VSMCs to ECs is suppressed by an shRNA against hnRNPA2B1. We also found that hnRNPA2B1 is up-regulated during neointima formation and hnRNPA2B1 inhibition accelerates re-endothelialization and attenuates neointima formation following carotid injury. To conclude, the transfer of exosomal miR-185 from VSMCs to ECs is controlled by hnRNPA2B1 and impairs re-endothelialization after vascular injury. These findings further the understanding of exosomal-mediated angiogenesis in restenosis and provide a basis for the development of exogenous therapies to prevent injury from vascular reconstruction.

## Data Availability Statement

The raw data supporting the conclusions of this article will be made available by the authors, without undue reservation.

## Ethics Statement

The animal study was reviewed and approved by the Experimental Animal Ethics Committee of Zhongshan Hospital Fudan University (Reference number: B2019-111R).

## Author Contributions

YS performed the experiments, acquired and analyzed the data, and contributed to the preparation of the manuscript. FL, DW, CF, XT, BG, ZS, ZD, and DG performed the experiments, acquired, and analyzed the data. JY and WF designed the research study, analyzed the data, and made the final approval of the version to be published. All authors read and approved the final manuscript.

## Conflict of Interest

The authors declare that the research was conducted in the absence of any commercial or financial relationships that could be construed as a potential conflict of interest.

## Supplementary Material

Supplementary Figure 1 qRT-PCR detection of miR-126, miR-17, miR-130a, miR-210, miR-221, miR-222, miR-92a, and miR-214 expression in exosomes derived from VSMCs in basal condition and from VSMCs exposed to 20 ng/mL PDGF for 24 h. ^∗^*P* < 0.05, ^∗∗^*P* < 0.01.
