# Supplementary figures and images for: Exosomal Transfer of miR-185 Is Controlled by hnRNPA2B1 and Impairs Re-endothelialization After Vascular Injury
Source: Front Cell Dev Biol. 2021 Apr 20;9:619444. doi: 10.3389/fcell.2021.619444 (PMC8093826; doi:10.3389/fcell.2021.619444)

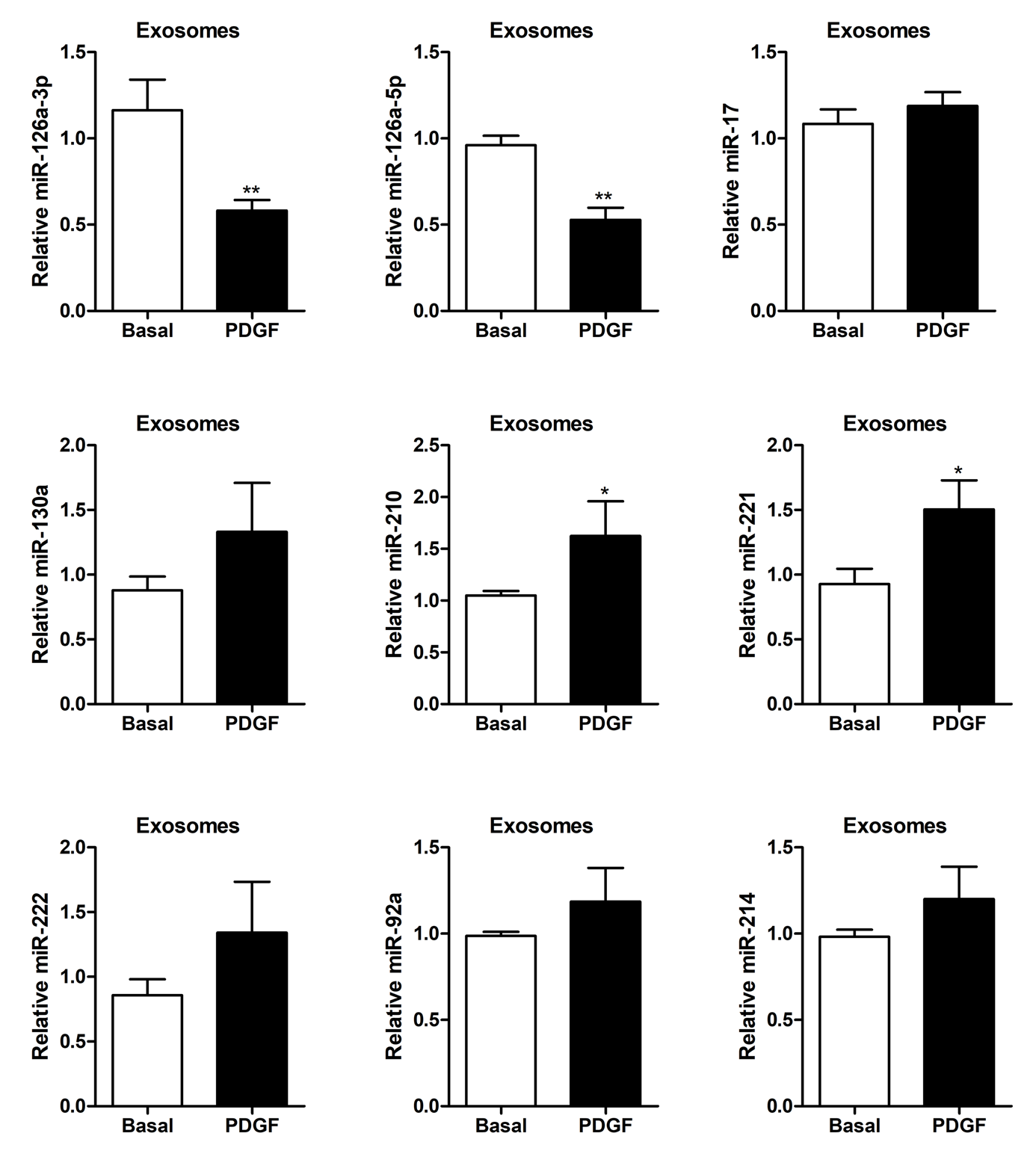

Supplement: Supplementary Figure 1 — qRT-PCR detection of miR-126, miR-17, miR-130a, miR-210, miR-221, miR-222, miR-92a, and miR-214 expression in exosomes derived from VSMCs in basal condition and from VSMCs exposed to 20 ng/mL PDGF for 24 h. ∗P < 0.05, ∗∗P < 0.01. [file Image_1.TIF]
